# Supplementary material for: Compositional Analysis of Four Kinds of Citrus Fruits with an NMR-Based Method for Understanding Nutritional Value and Rational Utilization: From Pericarp to Juice
Source: Molecules. 2022 Apr 16;27(8):2579. doi: 10.3390/molecules27082579 (PMC9031779; doi:10.3390/molecules27082579)
Supplement: Supplementary file 1 [file molecules-27-02579-s001.zip › molecules-1640355-supplementary.pdf]

Table S1. The quantitative results of the components identified from the citrus juices and pericarps in the different varieties

Components	Concentration (mM)							
	LJ[a]	DJ	OJ	PJ	LP	DP	OP	PP
1-Methylhistidine	NQ[b]	0.12±0.02[c]	0.16±0.03	0.06±0.01	0.29±0.12	0.42±0.07	0.18±0.05	0.33±0.07
2-Aminoisobutyrate	/[d]	/	/	/	0.08±0.03	0.04±0.02	0.05±0.02	0.07±0.01
2-Furoic acid	0.07±0.03	0.32±0.08	0.36±0.14	0.43±0.02	0.39±0.18	0.58±0.18	0.52±0.14	0.82±0.20
2-Ketobutyric acid	0.11±0.04	0.18±0.06	0.13±0.06	0.19±0.02	0.33±0.12	0.21±0.09	0.20±0.09	0.47±0.26
3-Hydroxybutyric acid	/	/	/	/	0.54±0.22	0.56±0.11	0.31±0.09	0.60±0.13
3-Methylphenylacetic acid	0.06±002	0.31±0.11	0.28±0.08	0.09±0.01	0.60±0.10	0.26±0.07	0.64±0.16	0.60±0.07
4-Hydroxybenzoic acid	NQ	NQ	NQ	NQ	0.06±0.02	0.06±0.01	0.15±0.06	0.10±0.03
4-Nitrophenol	/	/	/	/	0.05±0.01	0.03±0.01	0.07±0.02	0.05±0.02
7-Methylxanthine	/	/	/	/	0.05±0.01	0.07±0.02	0.07±0.01	0.09±0.03
Acetaldehyde	0.02±0.01	0.04±0.02	0.10±0.02	0.07±0.02	/	/	/	/
Acetic acid	0.06±0.01	0.92±0.22	1.27±0.39	0.30±0.03	/	/	/	/
Acetoin	0.10±0.03	0.17±0.05	0.10±0.04	0.11±0.01	0.04±0.02	0.04±0.01	0.06±0.02	0.05±0.01
Acetone	/	/	/	/	0.01±0.01	0.03±0.01	0.03±0.01	0.03±0.01
Adenosine	NQ	NQ	0.09±0.19	0.02±0.01	0.02±0.01	0.03±0.01	0.03±0.01	0.07±0.01
Alanine	0.11±0.03	1.37±0.61	1.32±0.59	2.21±0.26	0.53±0.15	0.39±0.11	0.54±0.20	1.35±0.36
Arginine	0.57±0.19	5.19±1.57	6.53±1.71	2.6±0.27	0.48±0.17	1.15±0.62	1.30±0.44	0.91±0.19
Ascorbic acid	0.77±0.37	2.00±0.64	2.77±0.92	1.72±0.67	/	/	/	/
Asparagine	57.45±51.16	8.68±2.39	4.79±3.05	18.82±2.08	11.45±5.99	2.83±3.76	0.99±0.41	3.63±0.90
Aspartate	1.27±0.57	6.24±3.05	9.80±1.91	4.15±0.47	1.34±0.59	1.66±1.00	1.01±0.47	1.85±0.40

Berberine	/	/	/	/	0.05±0.01	0.22±0.05	0.16±0.04	0.28±0.10
Betaine	0.17±0.03	0.72±0.24	0.59±0.24	0.76±0.09	0.13±0.04	0.71±0.27	0.42±0.19	0.48±0.13
Choline	0.09±0.03	0.38±0.08	0.20±0.07	0.27±0.03	0.18±0.05	0.89±0.39	0.47±0.24	0.45±0.24
Citric acid	3.6±1.03	28.37±13.19	43.22±15.47	59.36±6.55	6.75±3.69	1.16±0.42	1.03±0.43	1.45±0.88
Corticosterone	0.14±0.07	0.33±0.18	0.72±0.12	0.67±0.13	0.14±0.04	0.08±0.02	0.27±0.11	0.22±0.04
Cuminaldehyde	/	/	/	/	NQ	NQ	NQ	NQ
Ellagic acid	/	/	/	/	0.15±0.06	0.15±0.04	0.16±0.06	0.26±0.13
Erlose	0.42±0.09	1.93±0.74	1.69±0.53	2.10±0.35	/	/	/	/
Ethanol	5.56±2.05	6.69±3.14	4.45±1.90	2.65±0.32	0.50±0.16	0.38±0.08	0.54±0.21	0.54±0.26
Ethanolamine	/	/	/	/	0.18±0.04	0.55±0.25	1.54±0.94	0.59±0.13
Ethyl acetate	0.15±0.05	0.44±0.28	2.12±1.09	2.18±0.23	/	/	/	/
Flavone	/	/	/	/	0.06±0.02	0.08±0.04	0.08±0.03	0.10±0.03
Formic acid	NQ	NQ	NQ	NQ	NQ	NQ	NQ	NQ
Fructose	3.03±0.89	12.66±4.01	15.22±4.69	16.68±1.89	3.08±0.91	6.09±0.83	10.43±3.07	7.06±1.68
Fucose	0.78±0.18	1.95±0.60	2.29±0.48	3.40±0.46	0.17±0.05	0.84±0.48	0.33±0.11	0.34±0.09
Fumarate	/	/	/	/	0.09±0.03	0.08±0.04	0.08±0.03	0.10±0.03
Galactitol	/	/	/	/	2.04±0.50	3.51±0.78	3.77±1.15	5.12±1.40
Glutamic acid	0.75±0.30	7.82±2.18	5.71±2.59	1.20±0.14	1.62±0.34	2.57±0.66	10.44±3.66	7.01±1.78
Glutamine	2.37±0.56	3.37±0.99	2.83±1.05	4.78±0.51	1.54±0.48	0.99±0.21	0.99±0.30	1.38±0.23
Glycerol	/	/	/	/	1.88±0.41	3.51±0.60	4.75±0.73	3.16±0.54
Glycine	4.07±1.18	50.52±21.16	36.90±16.09	63.54±7.3	/	/	/	/
Gulonolactone	W[e]	W	W	W	0.94±0.26	1.38±028	1.67±0.47	1.49±0.39

Hesperetin	/	/	/	/	0.42±0.15	0.89±0.21	0.91±0.25	1.09±0.42
Hesperidin	0.05±0.02	0.04±0.04	0.13±0.04	0.53±0.05	0.37±0.08	6.10±3.74	1.47±0.42	1.85±0.45
Histamine	/	/	/	/	0.04±0.02	0.04±0.01	0.05±0.01	0.06±0.02
Hydroxymethylfurfural	/	/	/	/	NQ	NQ	NQ	NQ
Isobutyric acid	/	/	/	/	0.18±0.06	0.28±0.06	0.18±0.06	0.35±0.06
Isoleucine	0.12±0.02	0.18±0.06	0.12±0.08	0.21±0.02	0.19±0.06	0.13±0.04	0.17±0.05	0.27±0.09
Isovaleric acid	0.02±0.01	0.03±0.01	0.03±0.01	0.03±0.00	0.15±0.05	0.11±0.04	0.13±0.04	0.18±0.03
Lactose	1.34±0.32	2.01±0.66	3.43±1.08	3.66±0.78	0.79±0.24	0.91±0.20	1.09±0.29	1.45±0.37
Leucine	0.08±0.02	0.12±0.04	0.14±0.05	0.15±0.02	0.07±0.02	0.04±0.01	0.07±0.02	0.10±0.02
Limonin	0.02±0.03	0.25±0.11	0.62±0.16	0.41±0.05	0.32±0.06	0.27±0.10	0.42±0.15	0.48±0.12
Linalool	/	/	/	/	0.32±0.12	0.27±0.09	0.41±0.11	0.62±0.14
Lysine	0.25±0.06	2.26±0.49	2.02±0.41	0.72±0.08	0.29±0.08	0.56±0.25	0.88±0.27	0.68±0.18
Malic acid	1.21±0.47	3.67±1.03	7.15±3.47	3.91±1.07	1.46±0.57	3.23±1.30	3.10±1.34	3.92±1.5
Malonic acid	2.76±0.6	7.50±2.02	6.06±1.05	4.57±0.49	0.57±0.18	3.27±0.19	1.69±0.79	1.87±0.63
Maltotriose	/	/	/	/	0.22±0.18	0.41±0.26	0.44±0.17	0.44±0.11
Melezitose	/	/	/	/	0.20±0.06	0.50±0.25	0.39±0.12	0.57±0.12
Methanol	1.12±0.31	1.96±0.71	1.96±0.81	3.11±0.34	0.23±0.06	4.05±1.95	0.98±0.58	1.43±0.45
myo-Inositol	/	/	/	/	1.67±0.43	4.65±0.95	3.50±0.98	3.61±0.85
N,N-Dimethylglycine	0.37±0.07	0.24±0.12	0.55±0.13	4.21±0.58	0.07±0.03	0.04±0.03	0.02±0.01	0.03±0.01
Naringenin	0.12±0.04	0.41±0.16	0.58±0.10	0.72±0.12	/	/	/	/
Naringin	0.09±0.02	0.39±0.12	0.4±0.15	0.13±0.01	0.32±0.19	0.46±0.20	0.27±0.11	0.31±0.11
Neohesperidin	/	/	/	/	0.16±0.04	0.30±0.06	0.43±0.07	0.39±0.12

Neopterin	0.01±0.01	0.04±0.02	0.05±0.05	0.01±0.01	2.29±0.81	3.88±0.80	5.81±1.70	4.77±1.13
ortho-Hydroxyphenylacetic	/	/	/	/	2.45±0.48	5.62±1.20	4.44±0.98	3.65±0.64
para-Aminobenzoic acid	/	/	/	/	0.11±0.03	0.16±0.06	0.19±0.06	0.23±0.06
p-Cresol	0.02±0.01	0.06±0.02	0.08±0.03	0.03±0.01	/	/	/	/
Phenindione	/	/	/	/	0.02±0.01	0.02±0.01	0.02±0.01	0.03±0.01
Phenylalanine	0.03±0.02	0.29±0.06	0.22±0.05	0.16±0.01	0.16±0.03	0.33±0.15	0.38±0.08	0.36±0.07
Proline	2.35±0.81	18.49±6.49	17.42±6.05	11.94±1.64	0.26±0.07	0.44±0.09	0.57±0.17	0.47±0.07
Quinic acid	/	/	/	/	2.55±1.37	5.63±1.09	2.00±0.85	2.68±0.74
Raffinose	W	W	W	W	0.19±0.07	0.84±0.41	0.18±0.05	0.26±0.07
Rhamnose	0.08±0.03	0.20±0.07	0.22±0.06	0.53±0.01	0.72±0.23	2.49±0.68	0.24±0.09	1.01±0.35
scyllo-Inositol	0.97±0.21	2.52±0.92	2.1±0.89	2.12±0.24	/	/	/	/
Sebacic acid	/	/	/	/	0.10±0.04	0.08±0.02	0.10±0.04	0.18±0.09
Sphingosine	/	/	/	/	0.09±0.03	0.13±0.03	0.22±0.05	0.30±0.08
Stigmasterol	NQ	0.02±0.01	0.02±0.01	NQ	0.05±0.03	0.01±0.00	0.02±0.03	0.11±0.10
Succinic acid	0.04±0.04	0.97±0.43	0.57±0.47	0.18±0.02	0.11±0.04	0.08±0.02	0.19±0.10	0.11±0.03
Succinimide	/	/	/	/	0.13±0.05	0.15±0.10	0.34±0.14	0.56±0.17
Sucrose	4.27±1.43	167.65±52.75	109.12±31.78	206.88±23.4	3.37±1.18	20.19±6.66	20.23±5.73	18.61±8.15
Synephrine	0.03±0.01	0.22±0.10	0.22±0.07	0.14±0.02	/	/	/	/
Taurine	/	/	/	/	3.97±0.89	11.68±1.68	7.06±1.80	6.68±1.55
Threonine	0.11±0.02	0.30±0.08	0.29±0.11	0.45±0.05	0.3±0.07	0.46±0.08	0.38±0.10	0.62±0.10
Tiglic acid	/	/	/	/	0.24±0.07	0.60±0.41	1.57±0.59	0.47±0.11
Trehalose	W	W	W	W	0.12±0.03	0.35±0.16	0.49±0.17	0.16±0.04

Trigonelline	NQ	NQ	NQ	NQ	NQ	NQ	NQ	NQ
Trimethylamine	/	/	/	/	0.03±0.01	0.04±0.03	0.01±0.01	0.03±0.02
Trimethylamine N-oxide	0.22±0.08	1.93±0.51	1.65±0.46	1.41±0.15	/	/	/	/
Uridine	0.07±0.05	0.18±0.11	0.46±0.08	0.30±0.08	/	/	/	/
Valine	0.09±0.02	0.29±0.09	0.26±0.11	0.26±0.03	0.09±0.02	0.10±0.05	0.11±0.03	0.18±0.04
Vitamin C	/	/	/	/	0.72±0.20	0.79±0.21	1.46±0.57	1.56±0.47
α-Amino-N-butyric acid	/	/	/	/	0.40±0.11	0.18±0.03	0.26±0.08	0.39±0.07
α-Aminoisobutyrate	0.02±0.01	0.04±0.02	0.02±0.02	0.05±0.01	/	/	/	/
α-Glucose	7.75±2.19	32.81±10.69	39.23±12.64	51.68±5.78	8.18±2.12	13.42±3.07	12.56±.368	13.25±3.07
α-Hydroxyisobutyric acid	/	/	/	/	0.20±0.07	0.12±0.06	0.09±0.02	0.19±0.03
α-Ketoglutaric acid	0.24±0.10	1.76±0.52	1.67±0.91	1.44±0.16	0.20±0.06	0.28±0.08	0.28±0.11	0.96±0.52
β-Alanine	/	/	/	/	0.71±0.21	1.35±0.42	0.71±0.27	1.10±0.19
β-Glucose	10.36±3.38	50.79±15.22	59.92±17.65	72.69±8.69	13.95±3.62	22.27±4.15	21.78±6.41	23.01±5.41
γ-Aminobutyric acid	/	/	/	/	0.61±0.13	0.90±0.34	0.87±0.33	1.34±0.34
γ-Terpinene	/	/	/	/	0.33±0.09	0.44±0.12	0.55±0.14	0.71±0.14

[a] LJ: lemon juice; DJ: dekopon juice; OJ: sweet orange juice; PJ: pomelo juice. LP: lemon pericarp; DP: dekopon pericarp; OP: sweet orange pericarp; PP: pomelo pericarp

[b] NQ: Not available for the quantitative analysis due to the low signal-to-noise ratio (<10)

[c] The quantitative results are given in the form of mean ± standard deviation, which are calculated from ten samples in the each group.

[d] “/”: Not detectable in this variety of citrus juice or citrus pericarp

[e] W: Not available for the quantitative analysis due to the interference signal of this component by the water peak

Table S2. The statistical analysis of the differential components in the citrus juice between different varieties.

Components	LJ-Others[a]				DJ-Others				OJ-Others				PJ-Othes			
	p[b]	r[c]	VIP[d]	FC[e]	p	r	VIP	FC	p	r	VIP	FC	p	r	VIP	FC
Ace[f]	/[g]	/	/	/	/	/	/	/	4.5×10^{-9}	0.85	1.28	3.5	/	/	/	/
Bet	9.0×10^{-6}	0.84	1.34	1.6	/	/	/	/	/	/	/	/	/	/	/	/
CA	1.8×10^{-10}	0.89	10.99	17.3	12.5×10^{-4}	-0.53	10.22	0.1	5.6×10^{-4}	-0.69	9.76	0.2	7.0×10^{-4}	-0.63	9.26	0.2
Eth	3.8×10^{-6}	0.93	2.41	4.6	/	/	/	/	/	/	/	/	1.3×10^{-6}	-0.59	2.30	0.2
Fru	1.4×10^{-3}	-0.56	1.03	0.8	/	/	/	/	4.3×10^{-10}	0.93	2.03	1.4	/	/	/	/
Gln	6.0×10^{-6}	0.95	1.02	2.5	/	/	/	/	/	/	/	/	/	/	/	/
Gly	1.1×10^{-18}	-0.96	4.04	0.2	1.6×10^{-4}	0.68	4.12	1.4	7.5×10^{-3}	0.52	4.11	1.3	2.7×10^{-5}	0.75	5.20	1.4
MA	/	/	/	/	/	/	/	/	1.8×10^{-3}	0.80	1.24	3.3	/	/	/	/
MLA	2.1×10^{-5}	0.91	1.18	1.7	/	/	/	/	/	/	/	/	2.2×10^{-13}	-0.81	1.96	0.4
Mol	/	/	/	/	/	/	/	/	/	/	/	/	3.9×10^{-8}	-0.64	1.51	0.8
DMG	/	/	/	/	/	/	/	/	/	/	/	/	1.4×10^{-8}	0.66	1.27	1.8
Pro	/	/	/	/	1.4×10^{-8}	0.66	1.27	1.8	8.6×10^{-7}	0.72	1.23	2.0	/	/	/	/
s-I	1.7×10^{-5}	0.89	1.52	3.0	/	/	/	/	/	/	/	/	1.1×10^{-9}	-0.73	1.67	0.2
Sur	3.1×10^{-29}	-0.94	3.90	0.1	8.8×10^{-7}	0.87	4.82	1.7	/	/	/	/	1.7×10^{-5}	0.61	4.42	1.6
TMAO	/	/	/	/	/	/	/	/	/	/	/	/	7.9×10^{-11}	-0.85	2.23	0.7
α-Glc	1.7×10^{-5}	-0.67	1.07	0.8	8.6×10^{-5}	-0.52	1.13	0.9	3.0×10^{-7}	0.59	1.86	1.3	/	/	/	/
β-Glc	7.0×10^{-5}	-0.74	1.25	0.7	/	/	/	/	2.7×10^{-6}	0.80	2.69	1.5	/	/	/	/

[a] LJ: lemon juice; DJ: dekopon juice; OJ: orange juice; PJ: pomelo juice; others: the other citrus juices

[b] p: The P-values were transformed from Student's t-test. The P values less than 0.05 were used as the cutoff values for the statistical significance.

[c] r: Correlation coefficients, positive and negative signs indicate positive and negative correlation in the concentrations, respectively. The cutoff value of correlation coefficient for the statistical significance was set as 0.50.

[d] VIP: Variable importance in projection. The VIP values at the top of 10% of all VIP scores were used as the cutoff values for the statistical significance.

[e] FC: Fold change, the concentration ratio between the pair-wise groups.

[f] Metabolite abbreviations, the detailed information could be obtained in Table 1.

[g] "/": Any one parameter in correlation coefficient, VIP value and P-value is unqualified for the statistical significance.

Table S3. The statistical analysis of the differential components in the citrus pericarp between different varieties

Components	LP-Others[a]				DP-Others				OP-Others				PP-Othes			
	p[b]	r[c]	VIP[d]	fc[e]	p	r	VIP	fc	p	r	VIP	fc	p	r	VIP	fc
Ala[f]	/[g]	/	/	/	/	/	/	/	1.1×10^{-5}	0.84	2.36	2.6	/	/	/	/
Asp	5.0×10^{-4}	0.81	2.53	5.2	/	/	/	/	/	/	/	/	/	/	/	/
Bet	1.1×10^{-9}	-0.60	1.83	0.3	/	/	/	/	/	/	/	/	8.7×10^{-4}	0.75	3.02	2.0
Cho	5.6×10^{-7}	-0.52	1.76	0.3	/	/	/	/	/	/	/	/	1.6×10^{-3}	0.76	4.15	2.4
Cit	6.7×10^{-4}	0.81	2.94	6.0	/	/	/	/	/	/	/	/	/	/	/	/
ELA	/	/	/	/	3.4×10^{-3}	0.71	1.52	3.4	/	/	/	/	/	/	/	/
Fru	1.8×10^{-8}	-0.70	3.01	0.4	2.1×10^{-4}	0.85	4.74	2.0	/	/	/	/	/	/	/	/
Gol	7.8×10^{-7}	-0.64	1.17	0.6	/	/	/	/	1.2×10^{-3}	0.70	1.68	1.5	/	/	/	/
Glu	/	/	/	/	9.7×10^{-5}	0.88	1.95	2.9	/	/	/	/	2.8×10^{-5}	-0.50	1.40	0.4
Glo	6.5×10^{-12}	-0.83	3.08	0.4	1.7×10^{-3}	0.71	3.56	1.6	/	/	/	/	/	/	/	/
Hrd	/	/	/	/	3.3×10^{-3}	-0.54	1.93	0.4	/	/	/	/	1.1×10^{-5}	0.95	3.08	7.7
MLA	3.3×10^{-9}	-0.63	1.66	0.3	/	/	/	/	/	/	/	/	1.9×10^{-4}	0.69	2.17	2.4
Mol	/	/	/	/	6.0×10^{-4}	-0.56	3.42	0.3	/	/	/	/	3.3×10^{-4}	0.82	4.95	4.7
m-I	3.2×10^{-10}	-0.77	1.18	0.5	/	/	/	/	/	/	/	/	/	/	/	/
NPr	5.0×10^{-12}	-0.76	1.94	0.3	8.9×10^{-4}	0.81	1.98	1.8	/	/	/	/	/	/	/	/
o-HPA	4.7×10^{-10}	-0.74	2.50	0.5	/	/	/	/	/	/	/	/	/	/	/	/
QA	/	/	/	/	2.3×10^{-4}	-0.63	1.49	0.5	/	/	/	/	8.7×10^{-6}	0.72	1.72	2.2
Rha	/	/	/	/	1.8×10^{-6}	-0.67	1.54	0.2	/	/	/	/	3.1×10^{-5}	0.92	2.08	4.6
Sum	/	/	/	/	/	/	/	/	6.9×10^{-6}	0.87	2.03	2.8	/	/	/	/

Sur	4.8×10^{-14}	-0.88	4.86	0.2	/	/	/	/	/	/	/	/	/	/	/	/
Tau	3.0×10^{-8}	-0.73	1.41	0.5	/	/	/	/	/	/	/	/	/	/	/	/
α-Glc	7.1×10^{-5}	-0.69	1.80	0.7	/	/	/	/	/	/	/	/	/	/	/	/
β-Glc	4.7×10^{-5}	-0.67	3.38	0.7	/	/	/	/	/	/	/	/	/	/	/	/

[a] LP: Lemon pericarp; DP: Dekopon pericarp; OP: sweet orange pericarp; PP: Pomelo pericarp

[b] p: The P-values were transformed from Student's t-test. The P values less than 0.05 were used as the cutoff values for the statistical significance.

[c] r: Correlation coefficients, positive and negative signs indicate positive and negative correlation in the concentrations, respectively. The cutoff value of correlation coefficient for the statistical significance was set as 0.50.

[d] VIP: Variable importance in projection. The VIP values at the top of 10% of all VIP scores were used as the cutoff values for the statistical significance.

[e] FC: Fold change, the concentration ratio between the pair-wise groups.

[f] Metabolite abbreviations, the detailed information could be obtained in Table 1.

[g] "/": Any one parameter in correlation coefficient, VIP value and P-value is unqualified for the statistical significance
